# Welche mikrobiotaorientierten Therapien sind heute gesichert effektiv?

**DOI:** 10.1007/s00108-023-01471-8

**Published:** 2023-02-13

**Authors:** Michael Scharl

**Affiliations:** Klinik für Gastroenterologie und Hepatologie, Universitätsspital Zürich, Rämistrasse 100, 8091 Zürich, Schweiz

**Keywords:** Mikrobiotamodulation, Fäkale Mikrobiotatransplantation (FMT), Kolitis/*Clostridioides difficile*, Chronisch-entzündliche Darmerkrankungen, Nichtgastrointestinale Erkrankungen, Microbiota modulation, Fecal microbiota transplantation (FMT), Colitis/*Clostridioides difficile*, Inflammatory bowel diseases, Non-gastrointestinal diseases

## Abstract

**Hintergrund:**

Das Interesse an der Mikrobiota (dazu zählen Bakterien, Pilze und Viren) und an mikrobiotaorientierten Therapien ist in den letzten Jahren stetig gewachsen. Der Zusammenhang zwischen der Entstehung verschiedenster Erkrankungen und der Zusammensetzung der intestinalen Mikrobiota ist hier von besonderem Interesse. Insbesondere die Chancen, die eine gezielte Manipulation der Mikrobiotazusammensetzung in Therapieansätzen eröffnet, scheinen vielversprechend.

**Zielsetzung:**

Ziel dieses Übersichtsbeitrags ist es, die aktuelle Datenlage zu mikrobiotaorientierten Therapien zusammenzufassen sowie die mittels Studien nachweislich als effizient geltenden Therapieoptionen für bestimmte Erkrankungen aufzuzeigen.

**Ergebnisse:**

Die aktuelle Datenlage zur Effektivität mikrobiotabasierter Therapien variiert stark zwischen den untersuchten Erkrankungen. Während bestimmte Therapien in der Behandlung einiger Erkrankungen nachweisliche Erfolge erzielten, ist die Datenlage für andere Erkrankungen noch mangelhaft. So beträgt die Erfolgsrate bei der Behandlung einer *Clostridioides-difficile*-Kolitis mittels fäkaler Mikrobiotatransplantation 80–90 %.

**Schlussfolgerung:**

Die Behebung von Dysbiosen der intestinalen Mikrobiota kann eine Möglichkeit zur Behandlung der entsprechenden Erkrankungen darstellen. Mangels eines kausal-funktionellen Verständnisses und aufgrund der deskriptiven Natur der bisherigen Kenntnisse sind die Anwendungen bisher jedoch noch beschränkt. Die derzeit durchgeführten klinischen Studien zu Veränderungen und der Wichtigkeit unserer Darmmikrobiota könnten womöglich bald zu weiteren therapeutischen Optionen in der Behandlung verschiedener Erkrankungen führen.

## Abstract (en)

**Background:**

There is increasing interest in the microbiota (this includes bacteria, fungi and viruses) and microbiota-based therapies. The relationship between changes in the composition of the intestinal microbiota and the pathogenesis of various diseases is of specific interest. In particular, the possibilities offered by targeted manipulation of the microbiota composition as specific treatment approaches look promising.

**Objectives:**

This review article summarizes the current data on microbiota-based therapies as well as the evidence-based treatment options applicable for certain diseases.

**Results:**

Current data on the clinical effectiveness of microbiota-based therapies varies greatly between different diseases. While certain therapies proved successful in the treatment of some diseases, the data is still insufficient on their effectiveness in other diseases. So far, the most successful treatment in this context is fecal microbiota transplantation with a success rate of 80–90% for the treatment of *Clostridioides difficile* colitis.

**Conclusions:**

The correction of dysbioses of the intestinal microbiota could provide new possibilities for the treatment of diseases. However, due to the lack of a causal-functional understanding and the mainly descriptive knowledge to date, applications are still limited. The current clinical studies addressing the changes and the importance of intestinal microbiota could lead to new therapeutic options in the treatment of diverse diseases in the future.

Das Mikrobiom bzw. die Mikrobiota und ihre Rolle in der Pathogenese verschiedener Erkrankungen gewinnen seit einiger Zeit großes öffentliches Interesse. Das Interesse gilt insbesondere der Gesamtheit der Mikroorganismen (dazu zählen Bakterien, Pilze und Viren), die als „Mikrobiota“ bezeichnet wird; das „Mikrobiom“ dagegen bezeichnet die Erbinformation der Mikrobiota, also deren Genom. Die natürlicherweise im Menschen angesiedelten Bakterien, die den Hauptanteil der Mikrobiota ausmachen, wiegen etwa 2 kg und machen somit 2–4 % des gesamten Körpergewichts aus. In der Gesamtheit ist die Größe der Mikrobiota daher durchaus vergleichbar mit anderen Organen, so wiegt beispielsweise das menschliche Gehirn etwa 1,5 kg. Die sich mit der Beeinflussung der Zusammensetzung der intestinalen Mikrobiota eröffnenden Therapiemöglichkeiten sind äußerst interessant, stehen aber aktuell noch weitgehend am Beginn der Entwicklung.

## Bedeutung der humanen Mikrobiota

Die Zahl der verschiedenen Bakterienarten in der humanen Mikrobiota wird auf 300–1000 geschätzt. Die bisherigen Untersuchungen der Darmbakterien und ihrer Rolle im gesunden und erkrankten Organismus zeigen, dass die Existenz einer Bakterienart allein noch keine Rückschlüsse auf ihre Bedeutung oder Funktion zulässt. Einige Arten können in verschiedenen „Darmökosystemen“ sogar verschiedene metabolische Funktionen erfüllen.

Die Existenz einer Bakterienart allein lässt noch keine Rückschlüsse auf ihre Bedeutung im Darm zu

Zudem wird die Zusammensetzung der intestinalen Mikrobiota stark von Umwelteinflüssen, der Ernährung und verschiedenen Medikamenten beeinflusst. Daher ist die Zusammensetzung per se noch keine aussagekräftige Messgröße für vorhandene Erkrankungen oder Erkrankungsrisiken [1]. Dementsprechend können auf Basis der in großer Anzahl kommerziell angebotenen Mikrobiotaanalysen noch keine sinnvollen Therapieempfehlungen ausgesprochen werden.

## Möglichkeiten der Mikrobiotamodulation

Eine Möglichkeit, die Zusammensetzung der intestinalen Mikrobiota dennoch positiv zu beeinflussen, wurde mit der Gabe von Probiotika schon länger untersucht. Zudem existieren einzelne Medikamente, die tatsächlich Bakterien bzw. Pilze als therapeutisches Agens verwenden. Hier sind insbesondere *Escherichia* *coli* Nissle 1917, *Saccharomyces boulardii* oder *Enterococcus faecium* SF68 zu nennen. Eine weitere Methode zur Modifizierung der Darmmikrobiota ist die fäkale Mikrobiotatransplantation (FMT). Bei dieser Übertragung eines nicht weiter aufgetrennten Spenderstuhls in den Darm eines (erkrankten) Empfängers mittels Duodenalsonde, Einlauf oder Koloskopie – oder auch oral via Kapseln – werden bislang noch nicht genau verstandene günstige Veränderungen ausgelöst, die auf das Potenzial neuer Therapieansätze hinweisen.

## Erkrankungen mit veränderter Darmmikrobiota

In der Regel gilt eine vielfältige Mikrobiota als gesünder als eine Mikrobiota mit reduzierter Diversität. Zu den Erkrankungen, die mit einer Veränderung der Darmmikrobiota einhergehen, gehören

- gastrointestinale Erkrankungen:
  - *Clostridioides-difficile*-Kolitis [2],
  - chronisch-entzündliche Darmerkrankungen (CED; [3]),
  - Zöliakie,
  - Reizdarmsyndrom („irritable bowel syndrome“ [IBS]; [4]) und
  - Kolonkarzinom [5];
- hepatologische Erkrankungen [6, 7]:
  - alkoholische Lebererkrankung („alcoholic liver disease“ [ALD]),
  - nichtalkoholische Fettlebererkrankung („non-alcoholic fatty liver disease“ [NAFLD]),
  - autoimmune Lebererkrankungen und
  - hepatische Enzephalopathie;
- maligne Erkrankungen und Krebsimmuntherapien, beispielsweise
  - Melanome [8] und
  - Magenkarzinom;
- kardiovaskuläre und rheumatologische Erkrankungen:
  - Arteriosklerose und chronische Herzinsuffizienz [9],
  - rheumatoide Arthritis [10] und
  - entzündliche Kardiomyopathie [11];
- Erkrankungen des zentralen Nervensystems:
  - Depressionen,
  - Autismusspektrumstörungen,
  - Schizophrenie,
  - Angstzustände und
  - neurodegenerative Erkrankungen [12], beispielsweise Morbus Parkinson, Morbus Alzheimer und multiple Sklerose.

Eine therapeutische Modulation der intestinalen Mikrobiota kann bei einigen dieser Erkrankungen mittels FMT erreicht werden. Dabei ist die Wirkungsweise der FMT noch nicht vollständig geklärt. Ein therapeutischer Effekt kann auf die Übertragung lebender Bakterien oder aber auch auf die Übertragung von Metaboliten, die durch bestimmte Bakterien gebildet werden, zurückzuführen sein. Auch mit dem Infusat übertragene Bakteriophagen könnten eine Rolle spielen. Die verschiedenen Verabreichungswege der FMT scheinen sich in der Effektivität nicht wesentlich zu unterscheiden. Die FMT selbst gilt bei professioneller Durchführung und unter Berücksichtigung eines vorausgehenden Spenderscreenings zum Ausschluss übertragbarer Erkrankungen als sichere Methode.

Die FMT gilt bei professioneller Durchführung und vorherigem Spenderscreening als sichere Methode

**Figure 1:**
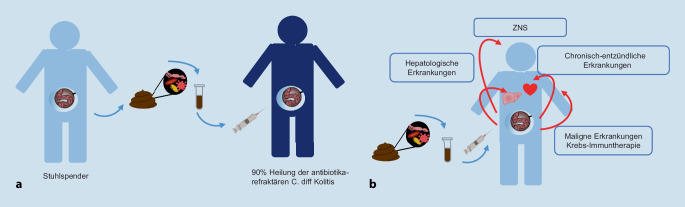

Zu den Erkrankungen, bei denen eine FMT positive Effekte zeigt, gehören als gastrointestinale Erkrankungen *C.-difficile*-Kolitis, chronisch-entzündliche Darmerkrankungen, IBS und intestinale „graft-versus-host disease“ (GvHD), zudem hepatische Erkrankungen. Eine weitere Erkrankung mit einem therapeutischen FMT-Effekt ist das therapierefraktäre maligne Melanom; auch für Autismus und weitere neuropsychiatrische Erkrankungen (hier bisher nur Fallberichte) sind Effekte gezeigt worden (Abb. 1).

## Fäkale Mikrobiotatransplantation zur Mikrobiotamodulation bei gastrointestinalen Erkrankungen

### *Clostridioides-difficile*-Kolitis

Seit 2013 und nach Abschluss der Studie von Els van Nood et al. [2] gilt die antibiotikarefraktäre *C.-difficile*-Kolitis als sinnvolle Indikation zur FMT. Als unerwünschte Nebenwirkungen können leichter Durchfall und abdominale Krämpfe am Tag der Infusion auftreten. Die Studie zeigte eine erhöhte fäkale Bakteriendiversität nach der Behandlung, insbesondere mit einer Zunahme der Bacteroidetes-Spezies und der *Clostridium*-Cluster IV und XIVa sowie einer Abnahme der Proteobacteria-Spezies. Die FMT ist mittlerweile durch zahlreiche Studien [13] und durch positive Ergebnisse im klinischen Alltag gut etabliert, zudem ist sie in europäischen Leitlinien sowie von der US Food and Drug Administration (FDA) in den USA als Therapiemethode der Wahl anerkannt. Die Behandlung der *C.-difficile*-Kolitis mittels FMT zeigt studienabhängige Erfolgsraten von 70 bis 95 %. Eine wiederholte FMT ist zwar möglich, jedoch selten nötig, da die Erfolgsrate einer einmaligen FMT via Koloskopie im Schnitt bei 88 % liegt [13].

### Chronisch-entzündliche Darmerkrankungen – Colitis ulcerosa

Eine erfolgreiche Behandlung der Colitis ulcerosa mittels FMT ist bei Verwendung eines „Superspender“-Infusats möglich [14]. Da die Bedingungen, die einen Superspender ausmachen, noch nicht geklärt sind, wird meist eine wiederholte, relativ aufwendige Therapie mit bis zu 40 Behandlungen verteilt über 8 Wochen durchgeführt, wobei ein Infusat verabreicht wird, das bis zu 7 Stuhlspenden kombiniert. Die Stuhlspenden konnten dabei zwar die Vielfalt der Mikrobiota nachhaltig vergrößern, dies ging aber leider nicht unbedingt mit einer relevanten klinischen Effektivität der Behandlung einher. Womöglich beeinflusst eine hohe Menge an *Candida* im Darm des FMT-Empfängers die Effektivität der FMT bei Patienten mit Colitis ulcerosa [15].

### Reizdarmsyndrom

Die Behandlung des IBS mittels FMT wurde in verschiedenen Studien mit unterschiedlichen Ergebnissen untersucht. Ein positiver Effekt in einigen Studien steht einem fehlenden Nutzen in anderen Studien gegenüber. Daher lässt sich für das IBS kein gesicherter Effekt durch eine FMT-Behandlung bestätigen [16].

### Intestinale „graft-versus-host disease“

Eine schwere GvHD kann als Folge einer allogenen hämatopoetischen Zelltransplantation zur Behandlung hämatologischer Malignome auch den Darm betreffen. Hier konnte in kleineren Fallserien gezeigt werden, dass die allogene FMT zur Linderung der GvHD-Symptome führen kann [17]. Parallel zur erfolgreichen Behandlung der intestinalen GvHD wurde bei 10 von 15 Studienteilnehmern die mikrobielle Diversität im Darm wiederhergestellt. Eine Bestätigung der Ergebnisse in größeren Studien steht noch aus.

## Fäkale Mikrobiotatransplantation zur Mikrobiotamodulation bei nichtgastrointestinalen Erkrankungen

### Hepatische Enzephalopathie.

Eine weitere belegte Indikation für die FMT ist die hepatische Enzephalopathie. Auch hier konnte im Rahmen klinischer Studien gezeigt werden, dass eine FMT nicht nur effektiv, sondern auch sicher ist, insbesondere bei Patienten mit Leberzirrhose und stark eingeschränkter Leberfunktion. In Anbetracht der in dieser Situation oftmals kritischen Lage des Patienten könnte eine FMT auch hier eine mögliche, effektive und sichere Therapieoption sein. Der Spenderstuhl wird hier von gesunden Spendern erhalten [18].

### Neurodegenerative und psychiatrische Erkrankungen.

Ein positiver Effekt der FMT konnte in klinischen Studien bisher lediglich für Patienten mit Autismus gezeigt werden. Zu *weiteren neuropsychiatrischen Erkrankungen* wurden lediglich einzelne Fallberichte oder Tierstudien veröffentlicht [19]. Eine Verbesserung der emotionalen Symptome von Patienten, die an Depression oder Angstzuständen leiden, könnte womöglich mit einer gezielten Mikrobiotabehandlung erreicht werden. Probiotika können einen Effekt auf den N. vagus haben, der jedoch individuell sehr variabel ist.

### Melanom.

Neuste Ergebnisse zweier Proof-of-concept-Studien konnten einen positiven Effekt der FMT bei immuntherapierefraktären Melanomerkrankungen aufzeigen. Den Patienten mit metastasiertem Melanom, die nicht auf eine Krebsimmuntherapie ansprachen, wurde mittels FMT der Stuhl von Spendern verabreicht, die sehr gut auf die Immuntherapie ansprachen. Dadurch konnten 30–40 % der Patienten von Nonrespondern zu Respondern der Therapie gemacht werden, das heißt, die Erkrankung sprach danach auf die Immuntherapie an [20, 21].

## Spezifische Mikroorganismen als therapeutisches Agens

### *Escherichiacoli* Nissle 1917

Dieses Bakterium ist zur Remissionserhaltung bei Patienten mit Colitis ulcerosa zugelassen und in der klinischen Routine gut etabliert. Im Rahmen klinischer Studien konnte nachgewiesen werden, dass es in dieser Indikation genauso effektiv wie Mesalazin ist [22].

### *Saccharomyces boulardii*

Die Hefe *Saccharomyces boulardii* wird insbesondere zur Behandlung akuter, beispielsweise bakteriell bedingter, Diarrhöen eingesetzt, aber auch zur Prävention der antibiotikaassoziierten Diarrhö oder der Reisediarrhö. Der Wirkmechanismus scheint hier insbesondere darin zu bestehen, dass *S. boulardii* – neben unter anderem immunologischen Effekten – zu einer Normalisierung der Dysbiose, also einer Regeneration des „normalen“ Darmmikrobioms beiträgt [23]. Daher ist auch eine Wirksamkeit bei antibiotikainduzierter Diarrhö denkbar. Dies wird unter anderem durch Daten aus klinischen Studien sowohl bei Kindern als auch bei Erwachsenen untermauert [24]. Hinsichtlich der Behandlung der akuten Diarrhö ist eine Wirksamkeit gerade bei pädiatrischen Patienten in Studien belegt. Daten einer 2020 publizierten Metaanalyse von Studien mit pädiatrischen Patienten zeigten, dass die Einnahme von *S. boulardii* bei akuter Diarrhö einerseits die Dauer der Symptomatik verkürzen und andererseits die Schwere der Symptomatik selbst sowie wohl auch die Hospitalisationsdauer verringern kann [25]. Allerdings muss angemerkt werden, dass das Evidenzniveau der in dieser Metaanalyse analysierten Studien insgesamt eher gering war. Als weitere Einsatzmöglichkeit von *S. boulardii* wird die Prävention der Reisediarrhö angesehen. Auch hier konnte die Wirksamkeit durch eine systematische Analyse der vorhandenen Studien im Rahmen einer Metaanalyse bestätigt werden [26].

### Weitere Probiotika

Auch andere Probiotika sind bei Indikationen wie der Prävention von antibiotikaassoziierten Diarrhöen oder Reisediarrhöen wirksam. Insbesondere *Lactobacillus rhamnosus* GG scheint eine gute Wirksamkeit in der Prävention der antibiotikaassoziierten Diarrhö zu haben, sowohl bei Kindern als auch bei Erwachsenen [27, 28]. In einer Übersichtsarbeit von Goodman et al. [28] konnte eine entsprechende Wirksamkeit auch für einige andere Mikroorganismen, vor allem Laktobazillen- und Bifidobakterienstämme gezeigt werden. Für die Einnahme von *Lactobacillus rhamnosus* ergab sich zudem ein Trend hinsichtlich einer Wirksamkeit zur Prävention der Reisediarrhö [26].

Zuletzt muss angemerkt werden, dass für zahlreiche andere probiotische Formulierungen, die entweder eine oder mehrere Spezies enthalten, in klinischen Studien positive Effekte auf die Gesundheit und/oder auf die Zusammensetzung der Darmmikrobiota gezeigt wurden.

## Fazit für die Praxis

Die veränderte Zusammensetzung der intestinalen Mikrobiota, die mit einer Reihe von Erkrankungen in Verbindung gebracht wird, deutet auf die Bedeutung unserer Darmmikrobiota hin. Eine Dysbiose kann in einigen Fällen durch eine fäkale Mikrobiotatransplantation (FMT) therapiert werden. So kann ein klinischer Erfolg dieses Ansatzes in der Therapie einer rezidivierenden *Clostridium-difficile*-Kolitis verbucht werden.

Zur Klärung des kausal-funktionellen Zusammenhangs zwischen Dysbiose und Krankheitsverlauf bei verschiedenen Erkrankungen werden derzeit viele klinische Studien durchgeführt. Ein Therapieansatz mittels gezielter Mikrobiotamodulation ist eine vielversprechende Zukunftsaussicht.

## References

[1] Gilbert JA, Blaser MJ, Caporaso JG (2018). Current understanding of the human microbiome. Nat Med.

[2] van Nood E, Vrieze A, Nieuwdorp M (2013). Duodenal infusion of donor feces for recurrent Clostridium difficile. N Engl J Med.

[3] Yilmaz B, Juillerat P, Oyas O (2019). Microbial network disturbances in relapsing refractory Crohn’s disease. Nat Med.

[4] Glassner KL, Abraham BP, Quigley EMM (2020). The microbiome and inflammatory bowel disease. J Allergy Clin Immunol.

[5] Tilg H, Adolph TE, Gerner RR (2018). The intestinal microbiota in colorectal cancer. Cancer Cell.

[6] Wang R, Tang R, Li B (2021). Gut microbiome, liver immunology, and liver diseases. Cell Mol Immunol.

[7] Duan Y, Llorente C, Lang S (2019). Bacteriophage targeting of gut bacterium attenuates alcoholic liver disease. Nature.

[8] Xavier JB, Young VB, Skufca J (2020). The cancer microbiome: distinguishing direct and indirect effects requires a systemic view. Trends Cancer.

[9] Witkowski M, Weeks TL, Hazen SL (2020). Gut microbiota and cardiovascular disease. Circ Res.

[10] Manasson J, Blank RB, Scher JU (2020). The microbiome in rheumatology: where are we and where should we go?. Ann Rheum Dis.

[11] Gil-Cruz C, Perez-Shibayama C, De Martin A (2019). Microbiota-derived peptide mimics drive lethal inflammatory cardiomyopathy. Science.

[12] Morais LH, Schreiber HL, Mazmanian SK (2021). The gut microbiota-brain axis in behaviour and brain disorders. Nat Rev Microbiol.

[13] Baunwall SMD, Lee MM, Eriksen MK (2020). Faecal microbiota transplantation for recurrent Clostridioides difficile infection: an updated systematic review and meta-analysis. EClinicalMedicine.

[14] Lam WC, Zhao C, Ma WJ (2019). The clinical and steroid-free remission of fecal microbiota transplantation to patients with ulcerative colitis: a meta-analysis. Gastroenterol Res Pract.

[15] Leonardi I, Paramsothy S, Doron I (2020). Fungal Trans-kingdom dynamics linked to responsiveness to fecal Microbiota transplantation (FMT) therapy in ulcerative colitis. Cell Host Microbe.

[16] Xu D, Chen VL, Steiner CA (2019). Efficacy of fecal microbiota transplantation in irritable bowel syndrome: a systematic review and meta-analysis. Am J Gastroenterol.

[17] van Lier YF, Davids M, Haverkate NJE (2020). Donor fecal microbiota transplantation ameliorates intestinal graft-versus-host disease in allogeneic hematopoietic cell transplant recipients. Sci Transl Med.

[18] Madsen M, Kimer N, Bendtsen F (2021). Fecal microbiota transplantation in hepatic encephalopathy: a systematic review. Scand J Gastroenterol.

[19] Vendrik KEW, Ooijevaar RE, de Jong PRC (2020). Fecal microbiota transplantation in neurological disorders. Front Cell Infect Microbiol.

[20] Baruch EN, Youngster I, Ben-Betzalel G (2021). Fecal microbiota transplant promotes response in immunotherapy-refractory melanoma patients. Science.

[21] Davar D, Dzutsev AK, McCulloch JA (2021). Fecal microbiota transplant overcomes resistance to anti-PD-1 therapy in melanoma patients. Science.

[22] Kruis W, Fric P, Pokrotnieks J (2004). Maintaining remission of ulcerative colitis with the probiotic Escherichia coli Nissle 1917 is as effective as with standard mesalazine. Gut.

[23] More MI, Swidsinski A (2015). Saccharomyces boulardii CNCM I-745 supports regeneration of the intestinal microbiota after diarrheic dysbiosis—a review. Clin Exp Gastroenterol.

[24] Szajewska H, Kolodziej M (2015). Systematic review with meta-analysis: Saccharomyces boulardii in the prevention of antibiotic-associated diarrhoea. Aliment Pharmacol Ther.

[25] Szajewska H, Kolodziej M, Zalewski BM (2020). Systematic review with meta-analysis: Saccharomyces boulardii for treating acute gastroenteritis in children—a 2020 update. Aliment Pharmacol Ther.

[26] McFarland LV, Goh S (2019). Are probiotics and prebiotics effective in the prevention of travellers’ diarrhea: a systematic review and meta-analysis. Travel Med Infect Dis.

[27] Szajewska H, Kolodziej M (2015). Systematic review with meta-analysis: Lactobacillus rhamnosus GG in the prevention of antibiotic-associated diarrhoea in children and adults. Aliment Pharmacol Ther.

[28] Goodman C, Keating G, Georgousopoulou E (2021). Probiotics for the prevention of antibiotic-associated diarrhoea: a systematic review and meta-analysis. BMJ Open.

